# A nomogram incorporating six easily obtained parameters to discriminate intrahepatic cholangiocarcinoma and hepatocellular carcinoma

**DOI:** 10.1002/cam4.1341

**Published:** 2018-02-23

**Authors:** Mengmeng Wang, Yuzhen Gao, Huijuan Feng, Elisa Warner, Mingrui An, Jian'an Jia, Shipeng Chen, Meng Fang, Jun Ji, Xing Gu, Chunfang Gao

**Affiliations:** ^1^ Department of Laboratory Medicine Shanghai Eastern Hepatobiliary Surgery Hospital Second Military Medical University Shanghai 200438 China; ^2^ Department of Surgery University of Michigan Medical School Ann Arbor 48109 Michigan; ^3^ Department of Epidemiology University of Michigan School of Public Health Ann Arbor 48109 Michigan

**Keywords:** Differential diagnosis model, hepatocellular carcinoma, intrahepatic cholangiocarcinoma, nomogram

## Abstract

Intrahepatic cholangiocarcinoma (ICC) and hepatocellular carcinoma (HCC) are the most prevalent histologic types of primary liver cancer (PLC). Although ICC and HCC share similar risk factors and clinical manifestations, ICC usually bears poorer prognosis than HCC. Confidently discriminating ICC and HCC before surgery is beneficial to both treatment and prognosis. Given the lack of effective differential diagnosis biomarkers and methods, construction of models based on available clinicopathological characteristics is in need. Nomograms present a simple and efficient way to make a discrimination. A total of 2894 patients who underwent surgery for PLC were collected. Of these, 1614 patients formed the training cohort for nomogram construction, and thereafter, 1280 patients formed the validation cohort to confirm the model's performance. Histopathologically confirmed ICC was diagnosed in 401 (24.8%) and 296 (23.1%) patients in these two cohorts, respectively. A nomogram integrating six easily obtained variables (Gender, Hepatitis B surface antigen, Aspartate aminotransferase, Alpha‐fetoprotein, Carcinoembryonic antigen, Carbohydrate antigen 19‐9) is proposed in accordance with Akaike's Information Criterion (AIC). A score of 15 was determined as the cut‐off value, and the corresponding discrimination efficacy was sufficient. Additionally, patients who scored higher than 15 suffered poorer prognosis than those with lower scores, regardless of the subtype of PLC. A nomogram for clinical discrimination of ICC and HCC has been established, where a higher score indicates ICC and poor prognosis. Further application of this nomogram in multicenter investigations may confirm the practicality of this tool for future clinical use.

## Introduction

Primary liver cancer (PLC), one of the most common solid tumor types, is a leading cause for cancer‐related death around the world 1, 2. Histologically, PLC can be roughly divided into three main subtypes: hepatocellular carcinoma (HCC), intrahepatic cholangiocarcinoma (ICC) and mixed hepatocellular–cholangiocellular carcinoma according to different cell origin 3. HCC is the major histopathologic subtype of PLC, accounting for more than 80% of the total intrahepatic primary malignancies 4, 5. Originating from the epithelial cells of the intrahepatic bile ducts, ICC ranks the second most prevalent primary hepatic malignancy, accounting for 10%~15% of PLC 6. Although ICC is less common than HCC, the incidence of ICC has been increasing drastically without clear and specific etiology in the United States during the past two decades, ranging from 0.72 to 0.88 per 100,000 7, 8, 9. In spite of recent advances in basic research and clinical trials, ICC reportedly bears a 5‐year survival of only about 30% 10.

As a malignant neoplasm, ICC and HCC frequently share common risk factors and clinical manifestations. However, as reported, attributing to different molecular characteristics and carcinogenic mechanisms, survival and prognosis of patients with ICC are more dismal than those with HCC 11. Although surgical resection is the first‐line treatment for HCC patients 12, 13, the best treatment option for ICC patients remains less defined, where common procedures differ between curative resection, chemotherapy, stereotactic radiotherapy, intra‐arterial therapy or multimodality treatment 14, 15, 16. As treatment modalities and clinical outcomes of ICC and HCC differ significantly, confidently discriminating between these two subtypes of PLC before making a medical decision has attracted more emphasis 17. Although magnetic resonance imaging (MRI) is a common emerging diagnostic procedure to discriminate the two subtypes, it still can be unfeasible in situations where patients cannot safely use an MRI or where the device is not easily accessible, and other methods such as ultrasound can still provide indistinguishable results 18, 19. As a result, alpha‐fetoprotein (AFP) and carbohydrate antigen 19‐9 (CA19‐9) are regarded as the optimal serum tumor markers for HCC and ICC, respectively. However, these two can also be unreliable, as the diagnostic sensitivity and specificity of these biomarkers are unsatisfactory, meaning better‐performing biomarkers are still needed 20, 21. Although many studies have been conducted to investigate the unique characteristics of ICC and find new differential diagnosis biomarkers or tools for distinguishing ICC from HCC, progress in clinical applications has still been limited 22, 23.

Given the lack of highly sensitive and specific predictive biomarkers and methods for ICC diagnosis, establishment of a predictive model that incorporates relevant factors can be another way to solve this issue. Nomograms, simple graphical systems, have been emerging in recent years and may be more accurate in preoperative diagnosis or prognostic evaluation than traditional methods for a variety of malignant neoplasms, including liver cancer 24, 25, pancreatic adenocarcinoma 26 and perihilar cholangiocarcinoma 27. In order to distinguish ICC from HCC before surgery without pathological validation, in this study, we aimed to establish and validate a differential diagnostic nomogram model for ICC and HCC based on the big data of the demographic characteristics and the results of routine laboratory tests.

## Materials and Methods

### Patient selection and study design

From January 2007 to January 2010, a total of 1614 eligible patients who received curative surgery for PLC at Eastern Hepatobiliary Surgery Hospital (EHBH, Shanghai, China) were recruited as the training cohort for the development of the nomogram model. In addition, 1280 patients with PLC at EHBH from January 2010 to January 2012 were enrolled in the validation cohort.

The inclusion criteria included the following: (1) histologic diagnosis was validated by post‐operative pathological examination, and (2) patient age fell between 40 and 60. Cause for exclusion included the following: (1) incomplete clinical information; (2) mixed hepatocellular–cholangiocellular carcinoma or other types of liver tumor were diagnosed; (3) patient history included other cancers; (4) preoperative treatment was administered.

All procedures involving human participants have been approved by the EHBH research ethics committee and have been performed in accordance with the 1964 Helsinki declaration and its later amendments or comparable ethical standards. Informed consent was obtained from all individual participants in advance.

### Follow‐up study

Apart from the two aforementioned retrospective study groups (training cohort and validation cohort), a small‐scale follow‐up study was conducted to compare the prognosis of the patients with ICC or HCC who received liver resection at our EHBH from January 2012 to January 2013. The enrolled patients were consecutively visited every 2 months for 2 years after the surgery and then every 3–6 months thereafter. Follow‐up discontinued at the time of cancer recurrence or cancer‐induced death. Overall survival (OS) was defined as the interval between operation and death or the time of latest visit. Recurrence‐free survival (RFS) was measured from hepatectomy to the date when recurrence/metastasis was detected.

### Laboratory measurement

Peripheral blood samples were collected after 12 h of fasting before surgery and were measured at the Department of Laboratory Medicine of EHBH. Patients underwent preoperative laboratory tests that included the following: aspartate aminotransferase (AST), alanine aminotransferase (ALT), *γ*‐glutamyl transferase (GGT), alkaline phosphatase (ALP), adenosine deaminase (ADA), total bile acid (TBA), total bilirubin (TBIL), direct bilirubin (DBIL), total protein (TP), prealbumin (PA), albumin (ALB), prothrombin time (PT), activated partial thromboplastin time (APTT), hepatitis B surface antigen (HBsAg), AFP, CA19‐9, carcinoembryonic antigen (CEA), complete blood count, etc. All results of these clinical laboratory tests were gathered as complete and comprehensive as possible.

### Statistical analysis

Baseline demographic information, results from the clinical laboratory tests and pathological studies, was collected and summarized in Table 1. Continuous variables are expressed as mean (SD) and compared using an unpaired student's *t*‐test or Mann–Whitney test. Categorical variables were compared using the Pearson's *χ*2 test or Fisher exact test. Survival curves were estimated using the Kaplan–Meier method, and differences were compared using the log‐rank test. Univariate logistic regression analysis was adopted to assess the differences in each potential factor in the training dataset for investigating independent risk factors for ICC. All variables associated with ICC at a significant level were the underlying candidates for stepwise multivariate analysis.

**Table 1:** Demographic information and clinicopathological characteristics of training and validation cohorts

Variables	Cohort, *n* (%)	
	Training (*n* = 1614)	Validation (*n* = 1280)
Age, mean (SD), years	50.71 (5.79)	50.41 (5.98)
Gender (Male, %)	1341 (83.1)	1064 (83.1)
Liver Cirrhosis		
Positive	772 (47.8)	652 (50.9)
Negative	842 (52.2)	628 (49.1)
Tumor size		
>3 cm	1282 (77.5)	1044 (81.6)
≤3 cm	332 (22.5)	236 (18.4)
Tumor Capsule		
Incomplete	1145 (70.9)	766 (59.8)
Complete	469 (29.1)	514 (40.2)
HBsAg		
Positive	1308 (81.0)	1029 (80.4)
Negative	306 (19.0)	251 (19.6)
AFP		
<20 ng/mL	801 (49.6)	626 (48.9)
20–400 ng/mL	377 (23.4)	285 (22.3)
>400 ng/mL	436 (27.0)	369 (28.8)
CA19‐9		
≥39 U/mL	434 (26.9)	361 (28.2)
<39 U/mL	1180 (73.1)	919 (71.8)
CEA		
≥10 *μ*g/L	69 (4.3)	52 (4.1)
<10 *μ*g/L	1545 (95.7)	1228 (95.9)
ALT		
≥45 U/L	501 (31.0)	437 (34.1)
<45 U/L	1113 (69.0)	843 (65.9)
AST		
≥40 U/L	512 (31.7)	471 (36.8)
<40 U/L	1102 (68.3)	809 (63.2)
GGT		
≥60 U/L	865 (53.6)	736 (57.5)
<60 U/L	749 (46.4)	544 (42.5)
ALP		
≥125 U/L	320 (19.8)	284 (22.4)
<125 U/L	1294 (80.2)	996 (77.8)
ADA		
≥7 U/L	869 (53.8)	942 (73.6)
<7 U/L	745 (46.2)	338 (26.4)
TP		
≥65 g/L	1331 (82.5)	1141 (89.1)
<65 g/L	283 (17.5)	139 (10.9)
ALB		
≥40 g/L	1124 (69.6)	886 (69.2)
<40 g/L	490 (30.4)	394 (30.8)
PA		
≥170 mg/L	1232 (76.3)	989 (77.3)
<170 mg/L	382 (23.7)	291 (22.7)
TBIL		
>20.52 *μ*mol/L	214 (13.3)	168 (13.1)
≤20.52 *μ*mol/L	1400 (86.7)	1112 (86.9)
DBIL		
>6.84 *μ*mol/L	378 (23.4)	270 (21.1)
≤6.84 *μ*mol/L	1236 (76.6)	1010 (78.9)
TBA		
≥12 *μ*mol/L	412 (25.5)	340 (26.6)
<12 *μ*mol/L	1202 (74.5)	940 (73.4)
PT		
≥12 sec	749 (46.4)	655 (51.2)
<12 sec	865 (53.6)	625 (48.8)
APTT		
≥37 sec	22 (1.4)	18 (1.4)
<37 sec	1592 (98.6)	1262 (98.6)
PLT		
≤100 × 10^3^/*μ*L	233 (14.4)	202 (15.8)
100–300 × 10^3^/*μ*L	1303 (80.7)	1021 (79.8)
≥300 × 10^3^/*μ*L	78 (4.8)	57 (4.5)
WBC		
≥4 × 10^3^/*μ*L	1380 (85.5)	1068 (83.4)
<4 × 10^3^/*μ*L	234 (14.5)	212 (16.6)

HBsAg, hepatitis B surface antigen; AFP, *α*‐fetoprotein; CA19‐9, carbohydrate antigen 19‐9; CEA, carcinoembryonic antigen; ALT, alanine aminotransferase; AST, aspartate aminotransferase; GGT, *γ*‐glutamyltransferase; ALP, alkaline phosphatase; ADA, adenosine deaminase; TP, total protein; ALB, albumin; PA, prealbumin; TBIL, total bilirubin; DBIL, direct bilirubin; TBA, total bile acid; PT, prothrombin time; APTT, activated partial thromboplastin time; PLT, platelet; WBC, white blood cell.

For nomogram construction, the most favorable model was screened and finally selected by multivariate logistic regression analysis using the Akaike's Information Criterion (AIC) as a stopping rule 28. The nomogram is based on transforming each variable's coefficient in the multivariate logistic regression into a 0‐ to 10‐point scale, proportionally. The effect of the variable with the highest *β* (absolute value) coefficient is assigned 10 points. Total scores, the sum of each included variable's point, are then converted to the risk probabilities of ICC presence. The concordance index (C‐index) was used to provide an estimate of the discrimination performance of the nomogram. A bootstrap method with 1000 resamples was implemented for model calibration to quantify the overfitting bias.

The diagnostic power of our nomogram model was assessed by a receiver operating characteristic (ROC) curve and the area under ROC curve (AUC), and the optimum cut‐off value for clinical use was determined by maximizing the Youden index (sensitivity + specificity − 1).

All the statistical tests were two‐tailed and *P *<* *0.05 was considered to indicate statistical significance. All analyses were performed by SPSS statistical software (SPSS Inc., Chicago, IL, version 22.0 for Windows) and the “rms” package of R, version 3.2.3.

## Results

### Demographic information and clinicopathological characteristics

In total, 2894 patients who received hepatic resection for PLC and met the study criteria were enrolled in this retrospective investigation. One thousand six hundred and fourteen and 1280 patients were included in the training cohort and validation cohort, respectively. As shown in Table 1, the clinicopathological and laboratory characteristics were similar between these two datasets. Histopathologically identified ICC was found in 401 (24.8%) and 296 (23.1%) patients in the training and validation cohort, respectively.

### Poorer prognosis of ICC patients

**Figure 1:**
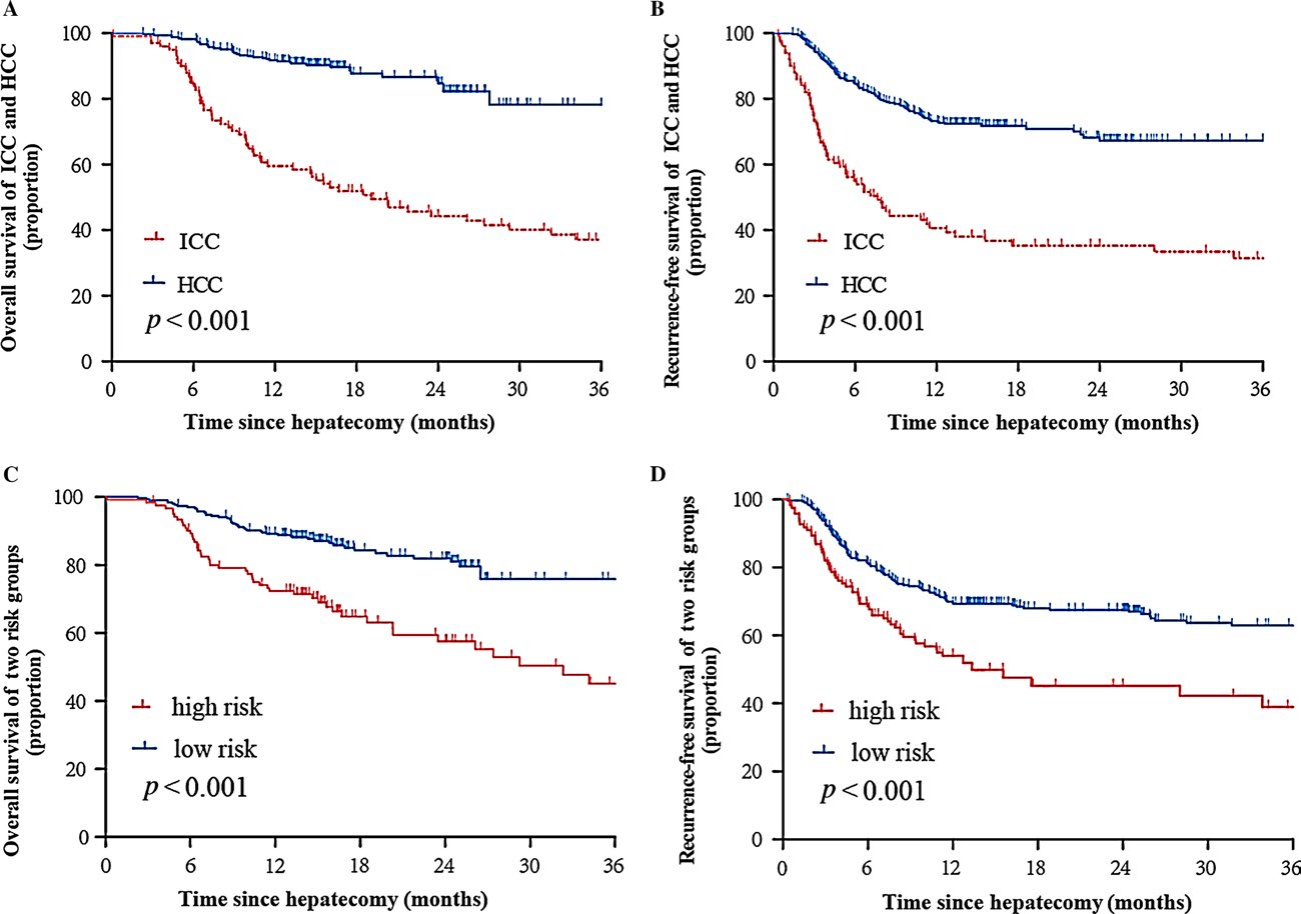
Overall survival (A) and Recurrence‐free survival (B) of ICC and HCC. Overall survival (C) and Recurrence‐free survival (D) of high‐risk group and low‐risk group. All the *P* values are <0.001. ICC, intrahepatic cholangiocarcinoma; HCC, hepatocellular carcinoma.

A follow‐up survey was implemented on 100 ICC and 330 HCC patients. The 1‐ and 3‐year OS rates of ICC were 59.5% and 37%, and those of HCC were 89.4% and 76.5%, respectively. The post‐operative 1‐ and 3‐year RFS of ICC were 39.3% and 31.5% and those of HCC were 72.3% and 67.3%, respectively. The log‐rank test showed that there were significant differences between ICC and HCC in the OS and RFS (*P *<* *0.01, Fig. 1A and B), indicating that ICC patients suffer a poorer prognosis than HCC patients.

### Establishment and validation of an ICC–HCC differential diagnosis nomogram model

The results of univariate logistic analysis are summarized in Table 2, and 18 candidate variables present significant differences between ICC and HCC in the training cohort. As listed in Table 3, Gender, HBsAg, AFP, CEA, CA19‐9, AST, ADA, TP, TBA, ALP and PT were independently associated with ICC based on the multivariate analysis.

**Table 2:** Univariate logistic regression analysis of ICC presence based on preoperative data in training Cohort (*n* = 1614)

Variables	Odds ratio (95% CI)	*P* value
Gender (Male vs. Female)	0.351 (0.267–0.462)	<0.01
Age (per year)	0.972 (0.953–0.991)	0.005
AFP (20–400 vs. <20, ng/L)	0.301 (0.221–0.408)	<0.01
(>400 vs. <20, ng/L)	0.285 (0.227–0.357)	<0.01
CA19‐9 (≥39 vs. <39, U/mL)	5.586 (4.369–7.141)	<0.01
CEA (≥10 vs. <10, mg/mL)	25.539 (11.563–47.918)	<0.01
Tumor Size (≥3 vs. <3, cm)	0.652 (0.578–0.735)	<0.01
Tumor Capsule (Incomplete vs. Complete)	12.477 (9.301–16.737)	<0.01
HBsAg (Positive vs. Negative)	0.099 (0.075–0.131)	<0.01
TBIL (≥20.52 vs. <20.52, *μ*mol/L)	0.897 (0.647–1.244)	0.515
DBIL (≥6.84 vs. <6.84, *μ*mol/L)	0.998 (0.765–1.303)	0.991
TBA (≥12 vs. <12, *μ*mol/L)	1.321 (1.009–1.729)	0.043
TP (≥65 vs. <65, g/L)	0.414 (0.288–0.594)	<0.01
ALB (≥40 vs. <40, g/L)	0.725 (0.562–0.936)	0.014
PA (≥170 vs. <170, mg/L)	0.752 (0.581–0.972)	0.03
ALT (≥45 vs. <45, U/L)	0.821 (0.640–1.053)	0.121
AST (≥40 vs. <40, U/L)	0.738 (0.574–0.949)	0.018
GGT (≥60 vs. <60, U/L)	1.461 (1.161–1.839)	0.001
ALP (≥125 vs. <125, U/L)	3.360 (2.593–4.353)	<0.01
ADA (≥7 vs. <7, U/L)	1.482 (1.178–1.866)	0.001
PT (≥12 vs. <12, sec)	0.604 (0.479–0.762)	<0.01
APTT (≥37 vs. <37, sec)	0.888 (0.326–2.423)	0.817

ICC, intrahepatic cholangiocarcinoma; AFP, *α*‐fetoprotein; CA19‐9, carbohydrate antigen 19‐9; CEA, carcinoembryonic antigen; HBsAg, hepatitis B surface antigen; TBIL, total bilirubin; DBIL, direct bilirubin; TBA, total bile acid; TP, total protein; ALB, albumin; PA, prealbumin; ALT, alanine aminotransferase; AST, aspartate aminotransferase; GGT, *γ*‐glutamyltransferase; ALP, alkaline phosphatase; ADA, adenosine deaminase; PT, prothrombin time; APTT, activated partial thromboplastin time.

**Table 3:** Multivariate logistic regression analysis of ICC presence based on preoperative data in the training cohort (*n* = 1614)

Variables	*β*	Odds ratio (95% CI)	*P* value
Gender (Male vs. Female)	0.831	2.295 (1.554–3.389)	<0.01
HBsAg (Positive vs. Negative)	−1.825	0.161 (0.111–0.234)	<0.01
TBA (≥12 vs. <12, *μ*mol/L)	−0.431	0.650 (0.444–0.952)	0.027
TP (≥65 vs. <65, g/L)	−0.644	0.525 (0.328–0.841)	0.007
AST (≥40 vs. <40, U/L)	−0.863	0.422 (0.287–0.620)	<0.01
ALP (≥125 vs. <125, U/L)	0.425	1.530 (1.023–2.289)	0.039
ADA (≥7 vs. <7, U/L)	0.728	2.071 (1.473–2.912)	<0.01
AFP (20–400 vs. <20, ng/L)	−0.990	0.372 (0.261–0.529)	<0.01
(>400 vs. <20, ng/L)	−2.312	0.099 (0.060–0.163)	<0.01
CEA (≥10 vs. <10, mg/mL)	1.808	6.100 (2.472–15.053)	<0.01
CA19‐9 (≥39 vs. <39, U/mL)	1.842	6.306 (4.440–8.956)	<0.01
PT (≥12 vs. <12, sec)	−0.336	0.714 (0.517–0.987)	0.042

ICC, intrahepatic cholangiocarcinoma; HBsAg, hepatitis B surface antigen; TBA, total bile acid; TP, total protein; AST, aspartate aminotransferase; ALP, alkaline phosphatase; ADA, adenosine deaminase; AFP, *α*‐fetoprotein; CEA, carcinoembryonic antigen; CA19‐9, carbohydrate antigen 19‐9; PT, prothrombin time.

**Figure 2:**
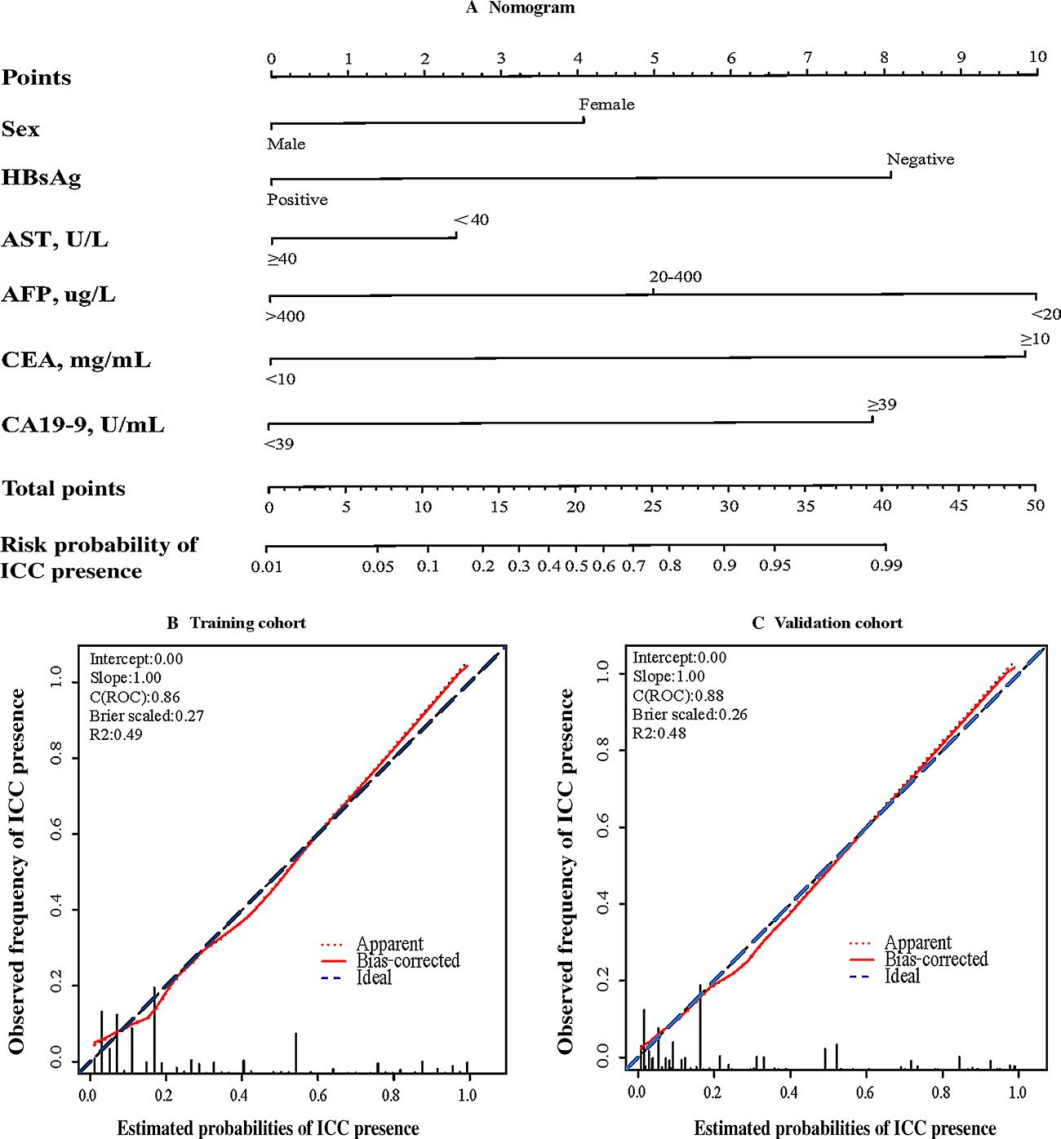
(A) Nomogram to discriminate ICC from HCC. To use the nomogram, match patient results for each parameter to a position on their corresponding axis, then draw a line to the Points axis at the top of the Nomogram to calculate the respective points for each parameter; finally, add the total points from all parameters, and draw a line from the Total Points axis to the Risk Probability axis at the bottom of the nomogram to determine ICC presence probabilities. (B) Validity of the discrimination efficacy in the training cohort (*n* = 1614). (C) Validity of the discrimination efficacy in the validation cohort (*n* = 1280). HBsAg, hepatitis B surface antigen; AST, aspartate aminotransferase; AFP, alpha‐fetoprotein; CEA, carcinoembryonic antigen; CA19‐9, carbohydrate antigen 19‐9; C, concordance index; ROC, receiver operating characteristic.

In accordance with AIC, the top six variables (Gender, HBsAg, CA19‐9, AFP, CEA and AST) were finally screened and integrated, which were mainly sorted based on *β* coefficient (absolute value) in the training cohort. A nomogram for discriminating ICC and HCC is demonstrated in Figure 2A. The model displays a C‐index of 0.86 (95% CI: 0.84–0.88), presenting a sufficient accuracy in differentiating ICC from HCC. The calibration plots revealed sufficient agreement between the nomogram and histopathologic examination results (Fig. 2B).

In the validation cohort, the C‐index was 0.88 (95% CI: 0.85–0.89), which also demonstrating that this nomogram model was sufficient in distinguishing ICC from HCC. There was also a sufficient calibration curve for the ICC presence probability estimation (Fig. 2C).

A nomogram score of 15 was determined as the optimal cut‐off value when the Youden index reached the maximum. As demonstrated in Table 4, the sensitivity, specificity, positive predictive value, and negative predictive value when using this model for differential diagnosis of ICC and HCC in the training cohort were 76.8%, 82.9%, 59.7% and 91.5%, and those of the validation cohort were 76.6%, 80.7%, 54.4% and 92%, respectively. The summary statistics of each candidate risk factor is listed in Table S1, which illustrates that no one variable alone can give satisfactory discrimination. Intriguingly, we also found that patients of either subtype who received a high‐risk score (>15, *n* = 122) based on this nomogram model suffered a more dismal prognosis than those who received a low‐risk score (≤15, *n* = 308) (Fig. 1C and D).

**Table 4:** Diagnostic efficacy of the nomogram model for estimating the presence of ICC

Variables	Value (95% CI)	
	Training Cohort (*n* = 1614)	Validation Cohort (*n* = 1280)
Cut‐off score (points)	15	15
Sensitivity (%)	76.80 (72.3–80.8)	76.60 (71.3–81.3)
Specificity (%)	82.90 (80.6–84.9)	80.70 (78.1–83.1)
Positive predictive value (%)	59.70 (55.3–63.9)	54.40 (49.5–59.3)
Negative predictive value (%)	91.50 (89.7–93.1)	92.00 (89.9–93.7)
Positive likelihood ratio	4.48 (3.91–5.13)	3.97 (3.44–4.58)
Negative likelihood ratio	0.27 (0.23–0.33)	0.28 (0.23–0.36)

## Discussion

Intrahepatic cholangiocarcinoma is a relatively rare cancer but with increasing incidence and mortality 29, 30. It shares several etiological risk factors and clinical presentations to HCC, but therapeutic strategies and prognoses differ significantly between these two major PLC subtypes, which make correct differentiation of ICC from HCC a major issue in clinical practice 19.

Intensive efforts have been devoted to assist clinicians in unveiling the differences between ICC and HCC. However, accurate discrimination of ICC from HCC before surgery or biopsy remains difficult when single or multiple masses are detected in the liver, or access to imaging methods remains limited. Several clinicopathological differences between patients with ICC and HCC have been elucidated and potential factors influencing survival have been identified, but a differential diagnosis model for ICC and HCC has still failed to be investigated for clinical use 31. New research focused on discriminating the two subtypes have redirected focus to their fundamental differences to assist in clinical diagnostics. In genetics, differentially expressed gene profiles and microRNAs patterns including miR‐21, miR‐31 and miR‐122 et al. in patients with ICC or HCC have been explored 32. During the past decades, the underlying metabolism reprogramming of cancer also led to ICC/HCC analysis. Comprehensive analysis and comparison of the transcriptomes and metabolomes of ICC and HCC have been reported, and distinct underlying carcinogenic mechanisms were studied, providing specific profile of genes and compounds, which might be useful in diagnosing ICC 33. Distinct fatty acid synthase in ICC and HCC specimens was decoded, and these findings supported some novel intervention approaches involving metabolism regulation 22. Additionally, histologic labels were investigated and some of them were potentially helpful in differential diagnosis of ICC and HCC. Immunohistochemical analyses revealed a significant higher expression level of sonic hedgehog (SHH) protein in ICC than in HCC 5. Li et al. 34 found that the CD79*α* (HM47/A9) antibody was effective in distinguishing between ICC and HCC. Other studies also demonstrated that anterior gradient 3 (AGR 3) and hepatocyte paraffin 1 were promising markers for discriminating ICC 17, 35. However, all these molecules were detected in tissue specimens with subpar diagnostic power, still limiting the feasibility of their use in clinical applications. These findings may help decipher the underlying molecular pathways and regulation mechanisms, but there is still a long way from basic research to clinical trials and practices 36. Imaging examination is also another important means for discrimination. However, the imaging characteristics of ICC might overlap with those of HCC in dynamic enhancement patterns in contrast‐enhanced ultrasound (CEUS) 19, 37. Recently, MRI‐based approaches have been developed to facilitate in accurate discrimination of ICC from HCC 23, 38. Nevertheless, costly high‐resolution equipment and experienced radiologists are not available in some developing areas, and many high‐risk patients are still ineligible for MRI use.

Nomograms can provide accurate risk evaluation and good discrimination characteristics that can facilitate the evidence‐based, individualized decision‐making 39, 40. We propose a nomogram model incorporating six comprehensive and easily obtainable preoperative variables (Gender, HBsAg, AST, AFP, CEA and CA19‐9) to discriminate ICC from HCC. It performed well in differentiating ICC from HCC, which were validated by the C‐index value of 0.86 and 0.88 in the training and validation cohorts, respectively. The optimal calibration curves demonstrated the coincidence between prediction and actual status. Among those factors, HBsAg, AST, AFP were negatively related to ICC, and female gender, CEA and CA19‐9 were positive parameters in this ICC‐differential model. High levels of AST and AFP were more common in HCC than in ICC, and HBV infection is one of the major causes of HCC 20. The nomogram model we have proposed can serve as an aid for clinical decision‐making and recruiting cases for randomized clinical trials including studies on neoadjuvant treatment for ICC. Figure 1A and B demonstrated poorer prognosis of ICC in our cohort compared with HCC, which was consistent with the literature and confirmed the necessity of an accurate discriminatory model.

To our knowledge, no nomogram model exists for differential diagnosis of ICC and HCC. In the present study, we have constructed and validated a simple and intelligible nomogram model based on a large‐scale study, which presented high accuracy (AUC >0.85) in a clinical setting. Our follow‐up assessments further demonstrated the nomogram's discrimination of high‐risk individuals correlated with poorer prognosis when compared with low‐risk individuals, independent of cancer subtype (Fig. 1C and D). However, some limitations of this study should be acknowledged. First of all, the data used in this study were obtained from a single institution, and multicenter validation is still necessary. Second, a prospective study is urgently needed to confirm the reliability of this model. Third, prognosis assessment value of this nomogram was observed in our small‐scale follow‐up study, and sample size should be enlarged for further validation.

## Conclusions

By combining six commonly assessed preoperative factors, a differential diagnostic model was established using nomogram analysis for optimal discrimination of ICC from HCC in a large‐scale, single‐center study and validated for accuracy. Further independent multicenter investigation is necessary to expand the validation for precision therapy and prognosis improvement.

## Conflict of Interest

None declared.

## Supporting information


**Table S1**. Differential diagnosis power of each candidate variable for discriminating ICC and HCC.
