# Supplementary material for: A nomogram incorporating six easily obtained parameters to discriminate intrahepatic cholangiocarcinoma and hepatocellular carcinoma
Source: Cancer Med. 2018 Feb 23;7(3):646–54. doi: 10.1002/cam4.1341 (PMC5852370; doi:10.1002/cam4.1341)
Supplement: Supplementary file 1 — Table S1. Differential diagnosis power of each candidate variable for discriminating ICC and HCC. [file CAM4-7-646-s001.docx]

Supplementary Table 1. Differential diagnosis power of each candidate variable for discriminating ICC and HCC

| **Variables** | **Cut-off** | **AUC** | **95% CI** | **Sensitivity (%)** | **Specificity (%)** |
| --- | --- | --- | --- | --- | --- |
| ADA, U/L | ≥7 | 0.599 | 0.548~0.651 | 22.190 | 90.600 |
| AFP, ng/ml | <21 | 0.768 | 0.742~0.793 | 64.090 | 76.500 |
| ALP, U/L | ≥125 | 0.584 | 0.534~0.635 | 46.380 | 77.410 |
| AST, U/L | <40 | 0.669 | 0.605~0.672 | 57.610 | 54.990 |
| CA19-9, U/L | ≥48 | 0.705 | 0.652~0.758 | 51.870 | 87.720 |
| CEA, ug/L | ≥10 | 0.554 | 0.498~0.609 | 24.190 | 94.150 |
| Gender | male | 0.627 | 0.575~0.679 | 29.430 | 87.220 |
| HBsAg | negative | 0.707 | 0.674~0.740 | 50.380 | 90.890 |
| PT, s | <12 | 0.588 | 0.555~0.621 | 52.000 | 64.430 |
| TBA, umol/L | <12 | 0.431 | 0.397~0.465 | 45.640 | 67.440 |
| TP, g/L | ≥65 | 0.558 | 0.510~0.607 | 60.100 | 68.100 |

Abbreviations: ICC, intrahepatic cholangiocarcinoma; HCC, hepatocellular carcinoma; AUC, area under curve; ADA, adenosine deaminase; AFP, α-fetoprotein; ALP, alkaline phosphatase; AST, aspartate aminotransferase; CA19-9, carbohydrate antigen 19-9; CEA, carcinoembryonic antigen; HBsAg, hepatitis B surface antigen; PT, prothrombin time; TBA, total bile acid; TP, total protein.
